# Prime labeling of graphs constructed from wheel graph

**DOI:** 10.1016/j.heliyon.2024.e23979

**Published:** 2024-01-05

**Authors:** Baha' Abughazaleh, Omar A. Abughneim

**Affiliations:** a Department of mathematics, Isra University, Amman, Jordan; b Department of mathematics, The University of Jordan, Amman, Jordan

**Keywords:** 05C78, Independence number, Wheels, Prime labeling, Prime graphs

## Abstract

A prime labeling of a simple undirected graph *G* is to assign unique integer labels from the set {1,2,...,|V(G)|} to each vertex such that any two adjacent vertices in the graph have labels that are relatively prime. Studying prime labeling in graphs can help us understand the structure and properties of graphs and prime labeling has potential applications in cryptography and network security. In this paper we investigate when some graphs that are constructed from wheels are prime graphs.

## 1 Introduction

An independent set of a finite simple undirected graph *G* is a set *A* of vertices in *G* where, within the set *A*, no pair of vertices are adjacent in *G*. The largest possible size of an independent set of vertices in *G* is known as the independence number of *G*, denoted by α(G). A bijective map *f* from the vertex set of *G* to the set {1,2,...,|V(G)|} is called a prime labeling (PL) of *G*, if f(a) and f(b) are relatively prime for any pair of adjacent vertices *a* and *b* in *G* and a graph *G* is called a prime graph (PG) if *G* has a PL. A PL defined by Entringer and was introduced by Tout et al. in [1]. Several families of graphs such as paths, stars, spiders and cycles are shown to be prime see [2], [3], [4]. This labeling technique has practical applications in network security and cryptography, as well as theoretical implications for understanding the structure and properties of graphs.

A wheel $W_{s}$ is a type of graph formed by joining a cycle $C_{s}$ to a single vertex. This single vertex is referred to as the apex vertex of $W_{s}$, while the vertices in the cycle are known as the rim vertices of $W_{s}$. It was shown in [5] that $W_{s}$ is a PG if and only if *s* is even. In [6], the PL of the graph formed by connecting apex vertices of $W_{s}$ and $W_{k}$ to a new vertex, where *s* and *k* are even integers, was examined. Also, the PL of a graph resulting from the identification of a rim vertex of $W_{s}$ with an endpoint of $P_{m}$ was investigated.

In this paper we generalize some of the results in [6]. We examine whether certain graphs obtained from wheels are PGs. For terms and concepts that have not been defined, readers are directed to consult the relevant references or sources for clarification in [7], [8], [9].

The motivation behind our work stems from a desire to explore and understand the properties of PGs related to wheels. We aim to investigate specific graph constructions involving wheels and paths, as well as wheels and cycles. By presenting lemmas and theorems related to these graph constructions and PL, we hope to provide a deeper understanding of the interplay between graph structures and PL. Our work aims to contribute to the existing body of knowledge in graph theory and potentially inspire further research in this topic.

## 2 Prime labeling of graphs constructed from wheel graph

In this section we will investigate the PL of graphs constructed from wheel graph. The following Lemma can be found in [10]. This Lemma puts some restrictions on the independence number of PGs.

Lemma 2.1 *[10]* *If G is a PG, then* $\alpha (G)\geq \left[\frac{\left|V(G)\right|}{2}\right]$ *.*

Theorem 2.2 *Let G be the graph formed by connecting a vertex of* $W_{m_{1}}$ *to a vertex of* $P_{m_{3}}$ *and a vertex of* $W_{m_{2}}$ *to a vertex of* $P_{m_{3}}$ *. If G is a PG, then* $m_{1}$ *and* $m_{2}$ *are even and* $m_{3}$ *is odd.*

Proof By Division Algorithm Theorem, we can write $m_{i}=2q_{i}+r_{i}$, where $0\leq r_{i}\leq 1$ for all i=1,2,3. Now

$$\left|V(G)\right|=m_{1}+m_{2}+m_{3}+2=2(q_{1}+q_{2}+q_{3}+1)+r_{1}+r_{2}+r_{3}.$$

It is clear that

(2.1) $$\alpha (G)=q_{1}+q_{2}+q_{3}+r_{3}$$

Since *G* is a PG. Then

(2.2) $$\alpha (G)\geq \left[\frac{\left|V(G)\right|}{2}\right]=q_{1}+q_{2}+q_{3}+1+\left[\frac{r_{1}+r_{2}+r_{3}}{2}\right].$$

If $m_{1}$ or $m_{2}$ is odd, then $1\leq r_{1}+r_{2}\leq 2$ and hence $\left[\frac{r_{1}+r_{2}+r_{3}}{2}\right]\geq r_{3}$. Substitute this relation in Inequality (2.2), we get

$$\alpha (G)\geq q_{1}+q_{2}+q_{3}+1+r_{3}.$$

But, this contradicts Equation (2.1). Thus $m_{1}$ and $m_{2}$ are even.

Also, if $m_{3}$ is even, then we can reduce Equation (2.1) to be

(2.3) $$\alpha (G)=q_{1}+q_{2}+q_{3}$$

and by Inequality (2.2)

$$\alpha (G)\geq q_{1}+q_{2}+q_{3}+1+\left[\frac{r_{1}+r_{2}}{2}\right]\geq q_{1}+q_{2}+q_{3}+1.$$

But, this contradicts Equation (2.3). Therefore $m_{3}$ is odd. □

In the upcoming theorem, a generalization of a result discussed in [6] is presented.

Theorem 2.3 *Let G be the graph formed by connecting the apex of* $W_{m_{1}}$ *to a vertex of* $P_{m_{3}}$ *and the apex of* $W_{m_{2}}$ *to a vertex of* $P_{m_{3}}$ *where* $m_{1}$ *and* $m_{2}$ *are even,* $m_{3}$ *is odd,* $(m_{1}+2,m_{1}+m_{2}+1)=1$ *and* $m_{1}+m_{2}+m_{3}+2$ *is prime. Then G is a PG.*

Proof Let $\left\{x_{1},x_{2},...,x_{m_{1}}\right\}$ and $\left\{y_{1},y_{2},...,y_{m_{2}}\right\}$ be the sets of successive rim vertices of $W_{m_{1}}$ and $W_{m_{2}}$ respectively, $x_{0}$ and $y_{0}$ be the apex vertices of $W_{m_{1}}$ and $W_{m_{2}}$ respectively and $\left\{z_{1},z_{2},...,z_{m_{3}}\right\}$ be the set of successive vertices of $P_{m_{3}}$. Define $f:V(G)⟶\{1,2,...,m_{1}+m_{2}+m_{3}+2\}$ as follows: $f(x_{i})=1+i$ for all $i=0,1,...,m_{1}$, $f(y_{j})=m_{1}+1+j$ for all $j=1,...,m_{2}$, $f(y_{0})=m_{1}+m_{2}+m_{3}+2$ and $f(z_{k})=m_{1}+m_{2}+k+1$. We want to show that for any two adjacent vertices *a* and *b* of *G*, we have (f(a),f(b))=1. To show that, we have the following cases:

1) Since $f(x_{0})=1$, then $f(x_{0})$ and $f(x_{i})$ are relatively prime for all $i=1,...,m_{1}$ and the label of the vertex of $P_{m_{3}}$ that is adjacent to $x_{0}$ in *G* is relatively prime with $f(x_{0})$.

2) Since $f(y_{0})=m_{1}+m_{2}+m_{3}+2$ is prime greater than $f(y_{j})$ and $f(z_{k})$ for all $j=1,...,m_{2}$ and for all $k=1,...,m_{3}$, then $f(y_{0})$ and $f(y_{j})$ are relatively prime for all $j=1,...,m_{2}$ and the label of the vertex of $P_{m_{3}}$ that is adjacent to $y_{0}$ in *G* is relatively prime with $f(y_{0})$.

3) For any two successive rim vertices of $W_{m_{1}}$, we have

$$(f(x_{i}),f(x_{i+1}))=(i+1,i+2)=1\text{ for all }i=1,...,m_{1}-1$$

and

$$(f(x_{m_{1}}),f(x_{1}))=(m_{1}+1,2)=1\text{ because }m_{1}+1\text{ is odd.}$$

4) For any two successive rim vertices of $W_{m_{2}}$, we have

$$(f(y_{j}),f(y_{l+1}))=(m_{1}+1+l,m_{1}+2+l)=1\text{ for all }l=1,...,m_{2}-1$$

and

$$(f(y_{m_{2}}),f(y_{1}))=(m_{1}+m_{2}+1,m_{1}+2)=1\text{ by assumption.}$$

5) For any two successive vertices of $P_{m_{3}}$, we have

$$(f(z_{k}),f(z_{k+1}))=(m_{1}+m_{2}+1+k,m_{1}+m_{2}+2+k)=1\text{ for all }k=1,...,m_{3}-1.$$

Thus *G* has a PL. □

According to Theorem 2.3 the following graph is a PG

**Figure:**
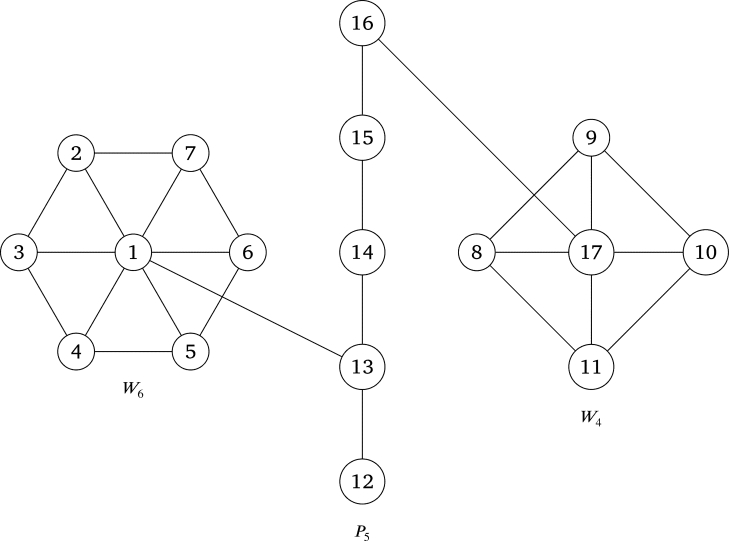

Theorem 2.4 *Suppose*$W_{m_{1}},W_{m_{2}}$*are wheels and*$P_{m_{3}}$*is a path with*$m_{1}$*and*$m_{2}$*are even,*$m_{3}$*is odd and*$C(P_{m_{3}})=\left\{v\right\}$*. Let G be the graph that is formed by connecting a rim vertex of*$W_{m_{1}}$*to a vertex of*$P_{m_{3}}$*, say u and joining the apex of*$W_{m_{2}}$*by a vertex of*$P_{m_{3}}$*. If*$m_{1}+m_{2}+m_{3}+2$*is prime,*$(m_{1}+2,m_{1}+m_{2}+1)=1$*and*$(m_{1}+1,m_{1}+m_{2}+\frac{m_{3}+1}{2}-d_{P_{m_{3}}}(u,v)+1)=1$*, then G is a PG.*

Proof Let $\left\{x_{1},x_{2},...,x_{m_{1}}\right\}$ and $\left\{y_{1},y_{2},...,y_{m_{2}}\right\}$ be the sets of successive rim vertices of $W_{m_{1}}$ and $W_{m_{2}}$ respectively, $x_{0}$ and $y_{0}$ be the apex vertices of $W_{m_{1}}$ and $W_{m_{2}}$ respectively and $\left\{z_{1},z_{2},...,z_{m_{3}}\right\}$ be the set of successive vertices of $P_{m_{3}}$. Without loss of generality assume $x_{m_{1}}$ is the rim vertex adjacent to *u* in *G* and $d_{P_{m_{3}}}(z_{1},u)\leq \frac{m_{3}-1}{2}$. So, *u* is on the geodesic between $z_{1}$ and *v*. Then

$$d_{P_{m_{3}}}(z_{1},u)=d_{P_{m_{3}}}(z_{1},v)-d_{P_{m_{3}}}(v,u)=\frac{m_{3}-1}{2}-d_{P_{m_{3}}}(v,u).$$

Thus $u=z_{\frac{m_{3}+1}{2}-d}$ where $d=d_{P_{m_{3}}}(v,u)$. Define $f:V(G)⟶\{1,2,...,m_{1}+m_{2}+m_{3}+2\}$ as the definition of *f* in the proof of Theorem 2.3. So,

$$(f(x_{m_{1}}),f(u))=(m_{1}+1,m_{1}+m_{2}+\frac{m_{3}+1}{2}-d+1)=1\text{ by assumption.}$$

Following the pattern of the proof for Theorem 2.3, it is evident that the labels of any other adjacent vertices in *G* are pairwise relatively prime. Thus *G* is a PG. □

According to Theorem 2.4 the following graph is a PG

**Figure:**
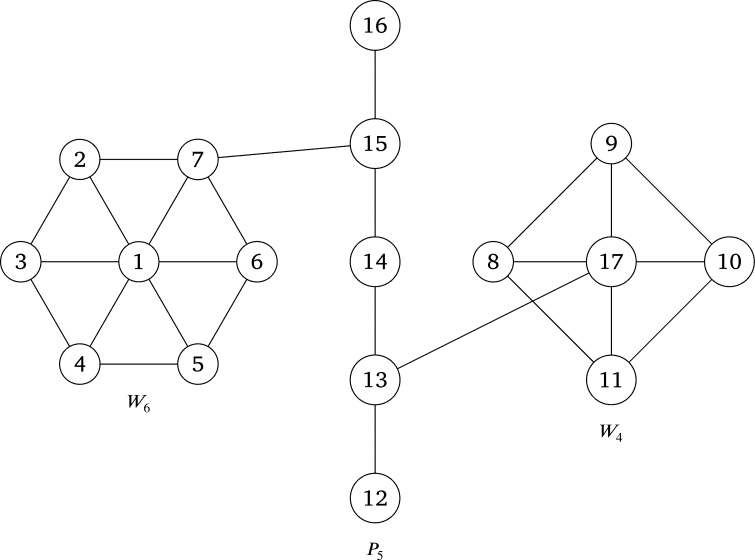

Corollary 2.5 *Suppose*$W_{m_{1}},W_{m_{2}}$*are wheels and*$P_{m_{3}}$*is a path with*$m_{1}$*and*$m_{2}$*are even,*$m_{3}$*is odd and*$C(P_{m_{3}})=\left\{v\right\}$*. Let G be the graph that is formed by connecting a rim vertex of*$W_{m_{1}}$*to a vertex of*$P_{m_{3}}$*, say u and joining the apex of*$W_{m_{2}}$*by a vertex of*$P_{m_{3}}$*. If*$m_{1}+m_{2}+m_{3}+2$*is prime,*$(m_{1}+2,m_{1}+m_{2}+1)=1$*and*$d_{P_{m_{3}}}(u,v)=\frac{m_{3}+1}{2}-2^{t}$*where t is an integer such that*$0\leq t\leq \log_{2}(\frac{m_{3}+1}{2})$*, then G is a PG.*

Proof Suppose $k=(m_{1}+1,m_{1}+m_{2}+\frac{m_{3}+1}{2}-d_{P_{m_{3}}}(u,v)+1)$. Then

$$k|(m_{1}+m_{2}+\frac{m_{3}+1}{2}-d_{P_{m_{3}}}(u,v)+1)-(m_{1}+1).$$

But

$$(m_{1}+m_{2}+\frac{m_{3}-1}{2}-d_{P_{m_{3}}}(u,v)+2)-(m_{1}+1)=m_{1}+m_{2}+\frac{m_{3}+1}{2}-(\frac{m_{3}+1}{2}-2^{t})+1-(m_{1}+1)=m_{2}+2^{t}.$$

So, $k|(m_{2}+2^{t})$. But $k|(m_{1}+1)$ and $m_{1}+1$ is odd. Thus *k* is odd and hence k=1. Using Theorem 2.4, we get *G* is a PG.

Also,

$$0\leq d_{P_{m_{3}}}(u,v)\leq \frac{m_{3}-1}{2}.$$

So,

$$0\leq \frac{m_{3}+1}{2}-2^{t}\leq \frac{m_{3}-1}{2}.$$

Thus

$$0\leq t\leq \log_{2}(\frac{m_{3}+1}{2}).$$

 □

Theorem 2.6 *Suppose* $W_{m_{1}},W_{m_{2}}$ *are wheels and* $P_{m_{3}}$ *is a path with* $m_{1}$ *and* $m_{2}$ *are even,* $m_{3}$ *is odd and* $C(P_{m_{3}})=\left\{v\right\}$ *. Let G be the graph that is formed by connecting a rim vertex of* $W_{m_{1}}$ *to a vertex of* $P_{m_{3}}$ *, say* $u_{1}$ *and joining a rim vertex of* $W_{m_{2}}$ *by a vertex of* $P_{m_{3}}$ *, say* $u_{2}$ *. If* $m_{1}+m_{2}+m_{3}+2$ *is prime,* $(m_{1}+2,m_{1}+m_{2}+1)=1$ *,* $(m_{1}+1,m_{1}+m_{2}+\frac{m_{3}+1}{2}-d_{P_{m_{3}}}(u_{1},v)+1)=1$ *and* $(m_{1}+2,m_{1}+m_{2}+\frac{m_{3}+1}{2}+{\left(-1\right)}^{q}d_{P_{m_{3}}}(u_{2},v)+1)=1$ *where*

$$q=\left\{ \begin{array}{c} 0\;\;\;\;\;\;\;\;\;\text{if}\;v\;\text{on the geodesic between}\;u_{1}\;\text{and}\;u_{2}\\ 1\;\text{if}\;v\;\text{is not on the geodesic between}\;u_{1}\;\text{and}\;u_{2} \end{array}\right.,\;\text{then }G\text{ is a PG}.$$

Proof Let $\left\{x_{1},x_{2},...,x_{m_{1}}\right\}$ and $\left\{y_{1},y_{2},...,y_{m_{2}}\right\}$ be the sets of successive rim vertices of $W_{m_{1}}$ and $W_{m_{2}}$ respectively, $x_{0}$ and $y_{0}$ be the apex vertices of $W_{m_{1}}$ and $W_{m_{2}}$ respectively and $\left\{z_{1},z_{2},...,z_{m_{3}}\right\}$ be the set of successive vertices of $P_{m_{3}}$. Without loss of generality assume $x_{m_{1}}$ is the rim vertex of $W_{m_{1}}$ adjacent to $u_{1}$ in *G*, $y_{1}$ is the rim vertex of $W_{m_{2}}$ adjacent to $u_{2}$ in *G* and $d_{P_{m_{3}}}(z_{1},u_{1})\leq \frac{m_{3}-1}{2}$. Thus $u_{1}=z_{\frac{m_{3}+1}{2}-d_{1}}$ where $d_{1}=d_{P_{m_{3}}}(v,u_{1})$. We want to show that $u_{2}=z_{\frac{m_{3}+1}{2}+{\left(-1\right)}^{q}d_{2}}$ where $d_{2}=d_{P_{m_{3}}}(v,u_{2})$. If *v* is on the geodesic between $u_{1}$ and $u_{2}$, then *v* is on the geodesic between $z_{1}$ and $u_{2}$. So,

(2.4) $$d_{P_{m_{3}}}(z_{1},u_{2})=d_{P_{m_{3}}}(z_{1},v)+d_{P_{m_{3}}}(v,u_{2})=\frac{m_{3}-1}{2}+d_{2}.$$

If *v* is not on the geodesic between $u_{1}$ and $u_{2}$, then *v* is not on the geodesic between $z_{1}$ and $u_{2}$. Then

(2.5) $$d_{P_{m_{3}}}(z_{1},u_{2})=d_{P_{m_{3}}}(z_{1},v)-d_{P_{m_{3}}}(v,u_{2})=\frac{m_{3}-1}{2}-d_{2}.$$

From Equation (2.4) and Equation (2.5), we get $d_{P_{m_{3}}}(z_{1},u_{2})=\frac{m_{3}-1}{2}+{\left(-1\right)}^{q}d_{2}$. Thus $u_{2}=z_{\frac{m_{3}+1}{2}+{\left(-1\right)}^{q}d_{2}}$. Define $f:V(G)⟶\{1,2,...,m_{1}+m_{2}+m_{3}+2\}$ as the definition of *f* in the proof of Theorem 2.3. So,

$$(f(y_{1}),f(u_{2}))=(m_{1}+2,m_{1}+m_{2}+\frac{m_{3}+1}{2}+{\left(-1\right)}^{q}d_{2}+1)=1\text{ by assumption.}$$

Following the pattern of the proof for Theorem 2.3 and Theorem 2.4, it is evident that the labels of any other adjacent vertices in *G* are pairwise relatively prime. Thus *G* is a PG. □

Corollary 2.7 *Suppose* $W_{m_{1}},W_{m_{2}}$ *are wheels and* $P_{m_{3}}$ *is a path with* $m_{1}$ *and* $m_{2}$ *are even,* $m_{3}$ *is odd and* $C(P_{m_{3}})=\left\{v\right\}$ *. Let G be the graph that is formed by connecting a rim vertex of* $W_{m_{1}}$ *to a vertex of* $P_{m_{3}}$ *, say* $u_{1}$ *and joining a rim vertex of* $W_{m_{2}}$ *by a vertex of* $P_{m_{3}}$ *, say* $u_{2}$ *. Also, assume that* $m_{1}+m_{2}+m_{3}+2$ *is prime,* $(m_{1}+2,m_{1}+m_{2}+1)=1$ *,* $d_{P_{m_{3}}}(u_{1},v)=\frac{m_{3}+1}{2}-2^{t}$ *and* $d_{P_{m_{3}}}(u_{2},v)={\left(-1\right)}^{q}\left(\frac{m_{3}+1}{2}-2^{s}\right)$ *where* $m=\left\{ \begin{array}{c} 0\;\;\;\;\;\;\;\;\;\text{if}\;v\;\text{on the geodesic between}\;u_{1}\;\text{and}\;u_{2},\\ 1\;\text{if}\;v\;\text{is not on the geodesic between}\;u_{1}\;\text{and}\;u_{2}, \end{array}\right.$ *and t is an integer such that* $0\leq t\leq \log_{2}(\frac{m_{3}+1}{2})$ *and s is an integer with* $\log_{2}(\frac{m_{3}+1}{2})\leq s\leq \log_{2}(m_{3})$ *whenever* m=0 *and* $0\leq s\leq \log_{2}(\frac{m_{3}+1}{2})$ *whenever* m=1 *. Then G is a PG.*

Proof Following the pattern of the proof for Corollary 2.5, we get $(m_{1}+1,m_{1}+m_{2}+\frac{m_{3}+1}{2}-d_{P_{m_{3}}}(u_{1},v)+1)=1$ and $0\leq t\leq \log_{2}(\frac{m_{3}+1}{2})$.

Suppose $l=(m_{1}+1,m_{1}+m_{2}+\frac{m_{3}+1}{2}+{\left(-1\right)}^{q}d_{P_{m_{3}}}(u_{2},v)+1)$. Then

$$l|(m_{1}+m_{2}+\frac{m_{3}+1}{2}+{\left(-1\right)}^{q}d_{P_{m_{3}}}(u_{2},v)+1)-(m_{1}+1).$$

But,

$$\left(m_{1}+m_{2}+\frac{m_{3}+1}{2}+{\left(-1\right)}^{q}d_{P_{m_{3}}}(u_{2},v)+1)-(m_{1}+1\right)$$

$$=m_{2}+\frac{m_{3}+1}{2}+{\left(-1\right)}^{q}{\left(-1\right)}^{q+1}\left(\frac{m_{3}+1}{2}-2^{s}\right)$$

$$=m_{2}+\frac{m_{3}+1}{2}-\left(\frac{m_{3}+1}{2}-2^{s}\right)=m_{2}+2^{s}.$$

Then $l|(m_{2}+2^{s})$ and so l=1, because *l* is odd. Thus by Theorem 2.6 *G* is a PG.

Also,

$$0\leq d_{P_{m_{3}}}(u_{2},v)\leq \frac{m_{3}-1}{2}.$$

So,

$$0\leq {\left(-1\right)}^{q+1}\left(\frac{m_{3}+1}{2}-2^{s}\right)\leq \frac{m_{3}-1}{2}.$$

Whenever m=0, we get

$$0\leq 2^{s}-\frac{m_{3}+1}{2}\leq \frac{m_{3}-1}{2}.$$

So,

$$\log_{2}(\frac{m_{3}+1}{2})\leq s\leq \log_{2}(m_{3}).$$

Whenever m=1, we get

$$0\leq \frac{m_{3}+1}{2}-2^{s}\leq \frac{m_{3}-1}{2}.$$

So,

$$0\leq s\leq \log_{2}(\frac{m_{3}+1}{2}).$$

 □

Remark 2.8 In Theorem 2.3, Theorem 2.4, Theorem 2.6 and Corollary 2.5, Corollary 2.7, if we join the apex of $W_{m_{1}}$ to a set of vertices of $P_{m_{3}}$ and the apex of $W_{m_{2}}$ to a set of vertices of $P_{m_{3}}$, then *G* remains a PG.

Theorem 2.9 *Let* t≥3 *,* $W_{m_{1}},W_{m_{2}},...$ *,* $W_{m_{t}}$ *are wheels and* $P_{s}$ *is a path. Then a graph G that is formed by connecting a vertex in each of the wheels* $W_{m_{1}},W_{m_{2}},...$ *,* $W_{m_{t}}$ *by a vertex in the path* $P_{s}$ *is not a PG.*

Proof By Division Algorithm Theorem, we can write $m_{i}=2q_{i}+r_{i}$, where $0\leq r_{i}\leq 1$ for all i=1,2,..,t. So

$$\left|V(G)\right|=\sum_{i=1}^{t}m_{i}+t+s\geq \sum_{i=1}^{t}(2q_{i}+r_{i})+s+3\geq \sum_{i=1}^{t}2q_{i}+s+3.$$

Suppose that *G* has a PL. Then

(2.6) $$\alpha (G)\geq \left[\frac{\left|V(G)\right|}{2}\right]\geq \sum_{i=1}^{t}q_{i}+\left[\frac{s+3}{2}\right].$$

But,

$$\alpha (G)=\sum_{i=1}^{t}q_{i}+\left\lceil \frac{s}{2}\right\rceil \leq \sum_{i=1}^{t}q_{i}+\left[\frac{s+1}{2}\right]<\sum_{i=1}^{t}q_{i}+\left[\frac{s+3}{2}\right].$$

This contradicts Inequality (2.6). Thus *G* does not have a PL. □

Theorem 2.10 *Let* t≥2 *,* $W_{m_{1}},W_{m_{2}},...$ *,* $W_{m_{t}}$ *are wheels and* $C_{s}$ *is a cycle. Then a graph G that is formed by connecting a vertex in each wheel* $W_{m_{1}},W_{m_{2}},...$ *,* $W_{m_{t}}$ *by a vertex in the cycle* $C_{s}$ *is not a PG.*

Proof Similar to the proof of Theorem 2.9, we have

$$\alpha (G)=\sum_{i=1}^{t}q_{i}+\left[\frac{s}{2}\right]<\sum_{i=1}^{t}q_{i}+\left[\frac{s+2}{2}\right]\leq \left[\frac{\left|V(G)\right|}{2}\right]$$

Because t≥2. Thus *G* is not a PG. □

In [6], it has been proven by the authors that if *s* is an even number, then the resulting graph resulting from the identification of a vertex from $W_{s}$ with an endpoint of $P_{t}$ is a PG. In [11], the authors proved that if s+t is prime, then the graph resulting from the identification of a rim vertex of $W_{s}$ with an end vertex of $P_{t}$ is a PG. In the following two theorems, we prove that if *s* and *t* are odd, then the graph resulting from the identification of a vertex of $W_{s}$ with a vertex of $P_{t}$ is not a PG. Also, we generalize the result in [11].

Theorem 2.11 *Let G be the graph resulting from the identification of a vertex of* $W_{s}$ *with a vertex of* $P_{t}$ *. If s and t are odd, then G is not a PG.*

Proof Suppose *s* and *t* are odd. Then by Division Algorithm Theorem, we can write s=2k+1 and t=2l+1. So, |V(G)|=n+m=2k+2l+2. But $\alpha (G)=k+l<\left[\frac{\left|V(G)\right|}{2}\right]=k+l+1$. Therefore, *G* is not a PG. □

Theorem 2.12 *Let G be the graph resulting from the identification of a vertex of* $W_{s}$ *with an end vertex of* $P_{t}$ *where* **s** *is odd and* **t** *is even.*

*If* (s,s+t)=1 *or* (t+1,s+t)=1 *, then G is a PG.*

Proof Let $x_{1},x_{2},...,x_{s}$ be the successive rim vertices of $W_{s}$, $x_{0}$ be the apex vertex of $W_{s}$ and $y_{1},y_{2},...,y_{t}$ be the vertices of $P_{t}$ such that $y_{t}$ is the vertex of $P_{t}$ that is identified with a vertex of $W_{s}$ in *G*. If we identify the apex vertex of $W_{s}$, then $y_{t}=x_{0}$. Otherwise assume $y_{t}=x_{s}$.

Firstly, if (s,s+t)=1, define f:V(G)⟶{1,2,...,s+t} as follows: $f(x_{i})=i+1$ for all i=0,1,...,s−1, $f(x_{s})=s+t$ and $f(y_{j})=s+j$ for all j=1,...,t−1. So, $(f(x_{1}),f(x_{s}))=(2,s+t)=1$, because s+t is odd. Also, $(f(x_{s-1}),f(x_{s}))=$ (s,s+t)=1 and $(f(y_{t-1}),f(y_{t}))=1$, because

$$f(y_{t-1})=s+t-1\text{ and }f(y_{t})=\left\{ \begin{array}{c} 1\;\;\;\;\;\;\text{if }y_{t}=x_{0}\\ s+t\text{ if }y_{t}=x_{s} \end{array}\right..$$

Now, if (t+1,s+t)=1, define f:V(G)⟶{1,2,...,s+t} as follows: $f(x_{0})=1$, $f(x_{i})=s+t-i+1$ for all i=1,2,...,s and $f(y_{j})=j+1$ for all j=1,...,t−1. So, $(f(x_{1}),f(x_{s}))=(s+t,t+1)=1$ and $(f(y_{t-1}),f(y_{t}))=1$, because

$$f(y_{t-1})=t\text{ and }f(y_{t})=\left\{ \begin{array}{c} 1\;\;\;\;\;\;\text{if }y_{t}=x_{0}\\ t+1\text{ if }y_{t}=x_{s} \end{array}\right..$$

In all cases, the labels assigned to any additional adjacent vertices in *G* are relatively prime. Therefore, *G* has a PL. □

According to Theorem 2.12 the following graph is a PG

**Figure:**
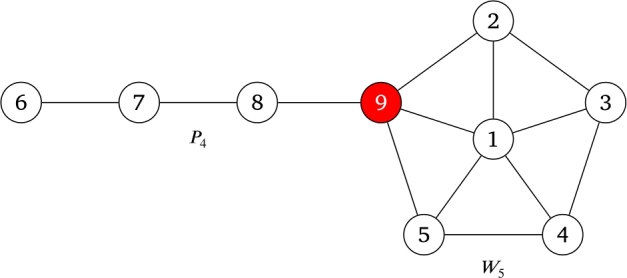

In the following two theorems, we study when the graph resulting from the identification of a vertex of $W_{s}$ with a vertex of $C_{t}$ is a PG.

Theorem 2.13 *Let G be the graph resulting from the identification of a vertex of* $W_{s}$ *with a vertex of* $C_{t}$ *where* **s** *and* **t** *are odd. Then G is not a PG.*

Proof The proof is similar to that of Theorem 2.11. □

Theorem 2.14 *Let G be the graph resulting from the identification of a vertex of* $W_{s}$ *with a vertex of* $C_{t}$ *. Then G is a PG, if one of the following statements holds*

*1)* s+t *is an odd prime.*

*2)* **s** *is even and whenever we identify a rim vertex of* $W_{s}$ *, then* (s+1,s+t)=1 *.*

Proof Let $x_{1},x_{2},...,x_{s}$ be the successive rim vertices of $W_{s}$, $x_{0}$ be the apex vertex of $W_{s}$ and $y_{1},y_{2},...,y_{t}$ be the vertices of $C_{t}$ such that $y_{1}$ is the vertex of $C_{t}$ that is identified with a vertex of $W_{s}$ in *G*. If we identify a rim vertex of $W_{s}$, suppose this vertex is $x_{1}$.

1) If s+t is an odd prime, then define f:V(G)⟶{1,2,...,s+t} as follows: $f(x_{0})=s+t$, $f(x_{i})=i$ for all i=1,...,s, and $f(y_{j})=s+j-1$ for all j=2,...,t. It is clear that *f* is a PL of *G*.

2) If **s** is even, then define f:V(G)⟶{1,2,...,s+t} as follows: $f(x_{0})=1$, $f(x_{i})=s-i+2$ for all i=1,...,s and $f(y_{j})=s+j$ for all j=2,...,t. Observe that

$$(f\left(x_{1}\right),f\left(x_{s}\right))=(s+1,2)=1\text{ because }s+1\text{ is odd}$$

If $y_{1}=x_{1}$, then

$$(f\left(y_{1}\right),f\left(y_{2}\right))=(s+1,s+2)=1$$

and

$$(f\left(y_{1}\right),f\left(y_{t}\right))=(s+1,s+t)=1.$$

If $y_{1}=x_{0}$, then $f\left(y_{1}\right)=1$. So,

$$(f\left(y_{1}\right),f\left(y_{2}\right))=1\text{ and }(f\left(y_{1}\right),f\left(y_{t}\right))=1.$$

Thus, *f* is a PL of *G*. □

In the following two theorems, we study when the graph resulting from the identification of a vertex of $W_{m_{1}}$ with a vertex of $W_{m_{2}}$ is a PG.

Theorem 2.15 *Let G be the graph resulting from the identification of a vertex of* $W_{m_{1}}$ *with a vertex of* $W_{m_{2}}$ *. If G is a PG, then m*_1_ *and m*_2_ *are even.*

Proof By Division Algorithm Theorem, we can write $m_{1}=2q_{1}+r_{1}$ and $m_{2}=2q_{2}+r_{2}$, where $0\leq r_{1},r_{2}\leq 1$. So,

$$\left|V(G)\right|=2q_{1}+2q_{2}+1+r_{1}+r_{2}.$$

But *G* is a PG. Then,

$$\alpha (G)=q_{1}+q_{2}\geq \left[\frac{\left|V(G)\right|}{2}\right].$$

So,

$$\left|V(G)\right|\leq 2q_{1}+2q_{2}+1.$$

Therefore, $r_{1}=r_{2}=0$. Thus $m_{1}$ and $m_{2}$ are even. □

Theorem 2.16 *Let G be the graph resulting from the identification of a vertex of* $W_{m_{1}}$ *with a vertex of* $W_{m_{2}}$ *, say this vertex is u, where* $m_{1}$ *and* $m_{2}$ *are even. Then G is a PG, if one of the following conditions holds*

*1) u is the apex vertex in* $W_{m_{1}}$ *and* $W_{m_{2}}$ *and* $(m_{1}+2,m_{1}+m_{2}+1)=1$ *.*

*2) u is the apex vertex in* $W_{m_{1}}$ *or* $W_{m_{2}}$ *and* $m_{1}+m_{2}+1$ *is prime.*

*3)* $(m_{1}+1,m_{1}+m_{2})=1$ *and* $m_{1}+m_{2}+1$ *is prime.*

Proof Let $\left\{x_{1},x_{2},...,x_{m_{1}}\right\}$ and $\left\{y_{1},y_{2},...,y_{m_{2}}\right\}$ be the sets of successive rim vertices of $W_{m_{1}}$ and $W_{m_{2}}$ respectively and $x_{0}$ and $y_{0}$ be the apex vertices of $W_{m_{1}}$ and $W_{m_{2}}$ respectively.

1) If *u* is the apex vertex in $W_{m_{1}}$ and $W_{m_{2}}$ and $(m_{1}+2,m_{1}+m_{2}+1)=1$, then define $f:V(G)⟶\{1,2,...,m_{1}+m_{2}+1\}$ as follows: f(u)=1, $f(x_{i})=i+1$ for all $i=1,...,m_{1}$ and $f(y_{j})=m_{1}+j+1$ for all $j=1,...,m_{2}$. Then

$$(f\left(x_{1}\right),f\left(x_{m_{1}}\right))=(2,m_{1}+1)=1$$

and

$$(f\left(y_{1}\right),f\left(y_{m_{2}}\right))=(m_{1}+2,m_{1}+m_{2}+1)=1.$$

So, *f* is a PL of *G*.

2) Let *u* be the apex vertex in $W_{m_{1}}$ or $W_{m_{2}}$ and $m_{1}+m_{2}+1$ is prime. Without loss of generality suppose *u* is the apex vertex in $W_{m_{1}}$. Case (1) if *u* is the apex vertex in $W_{m_{2}}$, then $(m_{1}+2,m_{1}+m_{2}+1)=1$ because $m_{1}+m_{2}+1$ is prime. So, by part (1) *G* is a PG. Case (2) if *u* is a rim vertex in $W_{m_{2}}$, then let $u=y_{1}$ and define $f:V(G)⟶\{1,2,...,m_{1}+m_{2}+1\}$ as follows: f(u)=1, $f(x_{i})=i+1$ for all $i=1,...,m_{1}$, $f(y_{0})=m_{1}+m_{2}+1$ and $f(y_{j})=m_{1}+j$ for all $j=2,...,m_{2}$. It is easy to check that *f* is a PL of *G*.

3) Suppose $(m_{1}+1,m_{1}+m_{2})=1$ and $m_{1}+m_{2}+1$ is prime. Case (1) if *u* is the apex vertex in $W_{m_{1}}$ or $W_{m_{2}}$, then by part (2) *G* is a PG. Case (2) if *u* is a rim vertex in $W_{m_{1}}$ and $W_{m_{2}}$, then let $u=x_{m_{1}}=y_{1}$ and define $f:V(G)⟶\{1,2,...,m_{1}+m_{2}+1\}$ as follows: $f(x_{0})=1$, $f(x_{i})=i+1$ for all $i=1,...,m_{1}$, $f(y_{0})=m_{1}+m_{2}+1$ and $f(y_{j})=m_{1}+j$ for all $j=2,...,m_{2}$. Then

$$(f\left(x_{1}\right),f\left(x_{m_{1}}\right))=(2,m_{1}+1)=1$$

and

$$(f\left(y_{1}\right),f\left(y_{m_{2}}\right))=(m_{1}+1,m_{1}+m_{2})=1.$$

Thus *f* is a PL of *G*. □

Conclusion 2.17 Our manuscript explored the prime labeling of graphs constructed from wheel graphs and their relationships with the independence number of prime graphs. Lemmas and theorems were presented, providing insights into the properties of prime graphs formed by joining vertices of wheels with paths and cycles. Specifically, it was shown that certain conditions on orders of the wheels, paths and cycles are necessary for a graph to be a prime graph. Establishing prime labeling of graphs constructed from wheel graph for other families of graphs different from paths and cycles is still open for further research.

## CRediT authorship contribution statement

**Baha' Abughazaleh:** Writing – review & editing, Writing – original draft, Visualization, Validation, Supervision, Software, Resources, Project administration, Methodology, Investigation, Formal analysis, Data curation, Conceptualization. **Omar A. Abughneim:** Writing – review & editing, Writing – original draft, Visualization, Validation, Supervision, Software, Resources, Project administration, Methodology, Investigation, Formal analysis, Data curation, Conceptualization.

## Declaration of Competing Interest

The authors declare no conflict of interest.

## Data Availability

No data was used for the research described in the article.

## References

[1] Tout A., Dabboucy A.N., Howalla K. (1982). Prime labeling of graphs. Nat. Acad. Sci. Lett..

[2] Fu H.L., Huang K.C. (1994). On prime labellings. Discrete Math..

[3] Gallian J.A. (2022). A dynamic survey of graph labeling. Electron. J. Comb..

[4] Pikhurko O. (2007). Trees are almost prime. Discrete Math..

[5] Lee S.M., Wui I., Yeh J. (1988). On the amalgamation of prime graphs. Bull. Malays. Math. Soc..

[6] Vaidya S.K., Prajapati U.M. (2011). Some results on prime and k-prime labeling. J. Math. Res..

[7] Agnarsson G., Greenlaw R. (2007).

[8] Burton D.M. (2009).

[9] Gross J.L., Yellen J. (2004).

[10] Youssef M.Z. (2000).

[11] Prajapati U.M., Gajjar S.J. (2014). Some results on prime labeling. Open J. Discrete Math..

